# Effect of Noradrenaline on the Virulence Properties of *Campylobacter* Species

**DOI:** 10.1155/2014/279075

**Published:** 2014-01-28

**Authors:** Sree V. Aroori, Tristan A. Cogan, Tom J. Humphrey

**Affiliations:** ^1^School of Veterinary Sciences, University of Bristol, Langford, Bristol BS40 5DU, UK; ^2^National Centre for Zoonosis Research, University of Liverpool, Leahurst Campus, Neston, Wirral CH64 7TE, UK

## Abstract

*Campylobacter* species cause a spectrum of illnesses in humans. The type of illness and the outcome is dependent on the virulence of the infecting pathogen strain and host immune status. Acute stress can seriously compromise host immunity and increase susceptibility to infection. Noradrenaline (NA) is a stress hormone. Several studies have shown that it stimulated growth and increased the pathogenicity of organisms including *E. coli* and *Campylobacter jejuni*. However, the effect of NA on other *Campylobacter* species is unknown. We have examined the effect of NA on growth rate, motility, invasion of T84 epithelial cells, and colonisation of chickens by diverse *Campylobacter* species. *Campylobacter* cultures grown with NA had reduced lag phases, increased growth rates, and higher final optical densities than controls. The motility of *Campylobacter* was also significantly increased in the presence of noradrenaline. Some of the *Campylobacter* strains tested also showed increased invasion of T84 epithelial cells, greater breakdown of tight junctions, and an enhanced potential to colonise chickens. Our results show that noradrenaline-induced enhancement of virulence of *Campylobacter* can influence the outcome of infection.

## 1. Introduction

*Campylobacter* species are the main cause of human bacterial gastroenteritis worldwide. *Campylobacter* is estimated to infect 1% of the population annually in the United Kingdom [1, 2]. *Campylobacter*, which commonly causes acute gastroenteritis, can also cause severe illnesses like Guillain-Barré and Miller Fisher syndromes, acute transverse myelitis, endocarditis, hepatitis, and myocardial injury in immunocompromised patients [3–6]. It is known that a disease outcome is dependent on the virulence of the infecting pathogen strain and host immune status [7]. Acute stress can seriously compromise host immunity and increase susceptibility to infection. Noradrenaline (NA) is a stress hormone. It is the principal neurotransmitter released in high quantities by the human enteric nervous system [8]. Bacterial pathogens entering the host through the gastrointestinal tract may be exposed to local high concentrations of NA [9]. Such exposure may contribute to the increased susceptibility of stressed hosts to infection. Several investigators have shown that NA increases the virulence and pathogenicity of bacteria by increasing growth [10, 11], by augmentation of host tissue attachment [12–16], or by involvement in cross kingdom signaling (Eukaryotic hormone causing a response in Prokaryotes) to activate transcription of virulence genes [17–19].

To date, studies on the effect of noradrenaline on *Campylobacter* species are very few. Noradrenaline was shown to increase the growth of *Campylobacter gracilis*. Growth rate, motility, and invasion of a single cell line by *C. jejuni* strain *11168* [20] were increased following exposure to noradrenaline. To find out if noradrenaline has a similar role on the virulence properties of other *Campylobacter* species, we examined the effect of NA on growth and motility of *Campylobacter* spp. We also examined the invasion of cultured T84 epithelial cells and their tight junction integrity and colonisation potential of three *Campylobacter* species in chickens.

## 2. Materials and Methods

### 2.1. Bacterial Strains and Culture Media

The *Campylobacter* strains used were *C. jejuni* 81116 [21], *C. jejuni M1* (environmental isolate), *C. coli* 1669 (pig kidney), *C. fetus fetus* 10842 (National Collection of Type Cultures, NCTC) [22], *C. jejuni 11168* (NCTC strain), and *C. coli* RM2228 (from Oxoid). The strains were cultured on Columbia agar supplemented with 5% citrated blood (BA) at 37°C for 48 hours in a microaerobic atmosphere (an environment in which the oxygen concentration is less than air) [20, 23].

The microaerobic atmosphere was created with Mart jars gassed with 5-6% O_2_, 6-7% CO_2_, and 2–4% H_2_ and the remaining with atmosphere N_2_, which was provided by Anoxomat system (Launch diagnostics Ltd., Kent, UK). For some experiments, the microaerobic atmosphere was provided by a MACS-MG 1000 anaerobic cabinet (DW scientific, Shipley, UK) (MAC-Cabinet) with 10% CO_2_, 5% O_2_, 2% H_2_, and 83% N_2_. All reagents unless otherwise stated were bought from Sigma Aldrich (Poole, Dorset, UK).

Three base media were used to prepare bacterial cultures for experiments: Mueller-Hinton (MH) broth (Oxoid, UK) and Dulbecco's Modified Eagles Medium (DMEM) (Invitrogen, UK) supplemented with 2 mM glutamine and 1% bovine serum (FBS) (PAA Labs, Germany).

### 2.2. Bacterial Growth Assays

The bacterial growth assays were performed with and without NA. The bacterial strains were grown on BA which was inoculated at 1 × 10^4^ colony forming units (cfu)/mL, into 1.5 mL volumes of DMEM with or without 160 *μ*M NA [20]. Cultures were grown in 200 *μ*L volumes in 100-well sterile covered honeycomb plates at 37°C for 48 hours in Microbiology Reader Bioscreen C (Labsystems, Helsinki, Finland). The optical densities (OD) were taken at 600 nm every 60 min and were plotted on a graph and those at stationary phase were compared using an unpaired *t*-test. All experiments were performed in triplicate on three separate occasions.

### 2.3. Measurement of Motility

A motility test, with and without NA, was performed on motility agar made of 0.4% (w/v) agar (Oxoid), 10 g/L peptone (Oxoid), 5 g/L Nacl (Merck), 3 g/L Beef extract (Oxoid), and 0.05 g/L of 2, 3, 5 triphenyl tetrazolium chloride (Sigma). To make NA-treated plates, 160 *μ*M NA was added to the motility agar before pouring into Petri plates. The agar was allowed to cool and plates were stabbed in the centre with the bacteria and incubated for 48 hours at 37°C. The test was done in triplicate and diameters of the motility halo were measured and compared using a paired *t*-test.

### 2.4. Invasion of *Campylobacter* into T84 Cells

The gentamicin resistance assay was used to measure the invasion of *Campylobacter* cells into T84 cells. The gentamicin resistance assay is based on principle that the gentamicin has limited ability to penetrate into eukaryotic cells [24]. The T84 human colonic adenocarcinoma cell line was obtained from European collection of cell cultures (ECACC, Health Protection Agency Culture Collection, Salisbury, UK) [25, 26]. The cells grow as monolayers and exhibit tight junctions and desmosomes between adjacent cells to prevent diffusion across the cell. The cells were grown at 37°C in 5% CO_2_ humidified atmosphere in DMEM supplemented with 10% foetal calf serum (FCS), penicillin and streptomycin at concentration of 200 units and 0.4 *μ*g/mL, respectively, and L-glutamine. A gentamicin protection assay was performed on confluent T84 cells grown for 10 days in a 12-well plate (Corning) at 37°C in a 5% CO_2_ incubator. *Campylobacter* species were grown for 48 h in DMEM + 10% FCS, with and without 160 *μ*M NA, in a microaerobic atmosphere. The inocula were adjusted to 0.1 OD at 600 nm and the monolayer infected at a multiplicity of infection (MOI) of 25 : 1 and incubated for 90 min at 37°C in a 5% CO_2_ incubator. The monolayer was washed with phosphate buffered saline (PBS) and 2 mL of gentamicin at 100 *μ*g/mL was added. Plates were then incubated at room temperature. After 2 h, the monolayer was washed in PBS and 2 mL of 1% Triton X-100 (Fluka, St. Louis, USA) was added. The monolayers were mixed well for 5 min, serially diluted, plated on BA plates, and incubated microaerobically for 48 h and the bacterial cells were counted. The results of the invasion assay were presented as the percentage of the input number of bacteria that survived the bactericidal action of gentamicin (invasion percentage). The tests were done in triplicate and the percentages of invasion were compared using a paired *t*-test.

### 2.5. The Effect of Noradrenaline on the Tight Junction Integrity of T84 Cells

A transwell model was used to observe the effects of NA on the ability of the *Campylobacter* strains to change tight junction integrity of T84 cells. T84 cells were grown on transwell inserts until transepithelial electrical resistance (TER) was above 300 Ω cm^2^ before *Campylobacter* was inoculated onto them. To make sure that NA on its own has no effect on TER, wells were incubated with NA for 48 h in every experiment and compared to uninfected controls. TER was recorded just before infection (T0). The apical wells were then infected with 30 *μ*L of bacteria (MOI of 10) or bacteria + 160 *μ*M of NA and incubated at 37°C in 5% CO_2_ for 48 h. The integrity of the monolayers was assessed by taking TER readings after 48 h (T48) and drop in TER was calculated by the following formula and compared by unpaired *t*-test:

(1) $$\begin{array}{c} \text{\%Drop TER}=100-\left(\frac{\text{T48}}{\text{T0}}\times 100\right). \end{array}$$

The transwells were then stained for the tight junction protein occludin. After infection, the transwells were fixed in ice-cold methanol at 4°C for 45 min, washed three times in PBS, and permeabilised in 1% Triton X-100 for 10 min at room temperature. Transwells were washed again three times in PBS and blocked with 5% human and goat serum for one hour at room temperature. This was followed by the addition of 100 *μ*L mouse anti-human occludin monoclonal antibody (Zymed, USA) at a concentration of 1 : 200 and incubated at 4°C overnight. The following day, the transwells were washed with PBS and 100 *μ*L goat anti-mouse FITC secondary conjugate (Southern Biotech, Alabama, USA) was added at a concentration of 1 : 100. This was then incubated at room temperature for one hour. The transwells were rewashed and filters cut out onto slides using a scalpel and mounted with a drop of vectashield (Vector laboratories, Peterborough, USA). Ten fields of view were visualised using a Leica DMRA fluorescence microscope with a Hamamatsu monochrome camera and digitised using Leica Q fluoro software and analysed by quantitative immunofluorescence using Macros on Image J software [27]. The average percentage area of occludin staining (green) was measured in the ten views and compared by an unpaired *t*-test.

### 2.6. The Effect of Noradrenaline Treatment on the Colonisation of Chickens by *Campylobacter*

Four groups of chicks at four weeks of age were housed at the School of Clinical Veterinary Science of the University of Bristol at a stocking density of 12.6 kg per m^2^ to meet the UK Home Office requirements. All animal experiments were conducted according to the requirements of the Animals (Scientific Procedures) Act 1986 and were approved by the local ethical review committee. Each group of birds was housed independently in separate rooms under strict conditions of biosecurity. The University of Bristol's Animal Services Unit biosecurity protocol was followed. This included the use of protective clothing (overalls, gloves, and disposable boot socks), throughout husbandry, and sampling. Protective clothing and footwear were changed and a disinfectant boot dip was used on every entry and exit from the rooms. The footwear was for dedicated use in the animal house and had not previously been worn outside. Feed and water was provided *ad libitum*. To ensure that the experimental birds were free from naturally occurring infection, faecal samples were collected before the challenge and tested for *Campylobacter* spp. Control birds were housed separately and samples were analysed at each postmortem examination to confirm the absence of *Campylobacter*.

*Campylobacter* spp. were grown for 48 h at 37°C in either CMH broth or this medium containing 160 *μ*M of NA, for NA-treated bacterial cells. Birds were given ~10^6^ viable cells of *C. jejuni M1*, grown either in the presence or absence of NA, by oral gavage. Forty-eight hours after infection a postmortem was performed and liver and caeca were collected for bacterial analysis. Caecal contents were removed into a universal tube, diluted 1 in 10 with saline, and vortexed to an even suspension. They were serially diluted to 10^−6^ and 100 *μ*L of each dilution was plated on charcoal cefoperazone deoxycholate agar (CCDA) and incubated at 37°C under microaerobic conditions for 48 h. Colonies were counted and the number of CFU/mL was determined by the following calculation (mean colonies per plate/100) × 1000 × dilution^−1^. Liver samples were homogenised with nine volumes of Exeter broth, 200 *μ*L plated on CCDA agar for a direct count. Six mL of the remaining homogenate was enriched in additional Exeter broth in a 37°C incubator. After 48 hours, the top layer was swabbed onto a CCDA plate and incubated in a microaerobic atmosphere for *Campylobacter* identification.

## 3. Results

### 3.1. Growth in the Presence of Noradrenaline

**Figure 1:**
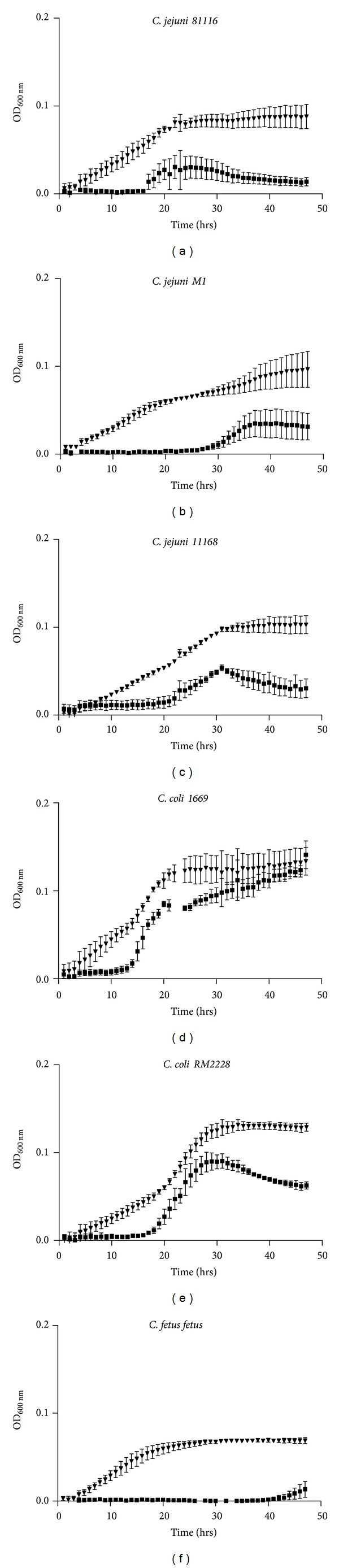
Growth of *C. jejuni 81116* (a), *C. jejuni M1* (b), *C. jejuni 11168* (c), *C. coli 1669* (d), *C. coli RM2228* (e), and *C. fetus fetus* (f) species taken over 48 h with readings every hour in the presence and absence of NA at 37°C. Results are the mean of three separate experiments, and bars represent standard error. The control group values are represented by solid squares and noradrenaline-treated group values are represented by solid triangle shape.

*Campylobacter* species were grown in the presence of 160 *μ*M noradrenaline for 48 hours at 37°C. The following results represent the average of three technical and three biological replicates. Optical density values at the start of stationary phase were compared by an unpaired *t*-test. All *Campylobacter* strains showed reduced lag phase and an increase in growth rate and the final optical density in the presence of noradrenaline (Figures 1(a)–1(f)). However, the reduction in lag phase and growth rate during exponential phase was much higher for *C. jejuni 81116* (*P* = 0.0135), *C. jejuni M1* (*P* = 0.029), *C. fetus fetus* (*P* = 0.0001), and *C. jejuni 11168* (*P* = 0.0006) in the presence of NA. Although there was higher reduction in lag phase and significant increase in final optical densities of NA-treated *C. coli* 1669 (Figure 1(d)) and *C. coli* RM2228 (Figure 1(e)), there was no change in growth rate during exponential phase. The mean ± SEM of three experiments was compared using an unpaired *t*-test and the significance values (*P* value) are shown in Table 1.

### 3.2. Motility in the Presence of NA

**Figure 2:**
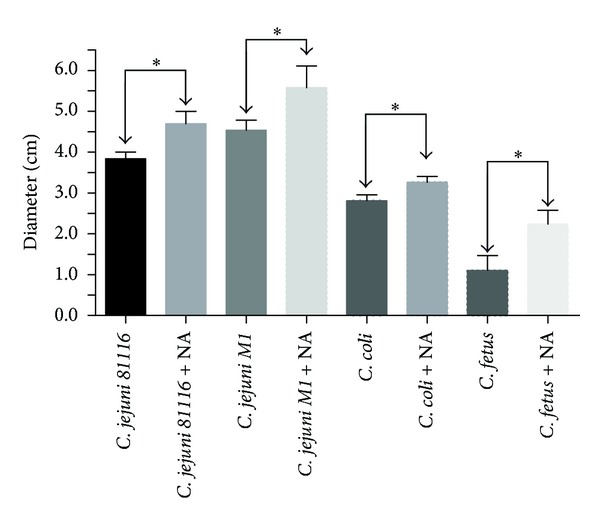
Motility of *C. jejuni 81116, C. jejuni M1, C. coli 1669*, and* C. fetus fetus* at 37°C. Size of the motility halo after 48 hours in the absence (black) or presence (white) of NA. The diameter of the halo was higher in the presence (white) of NA for all the species of *Campylobacter*. Star indicates *P* < 0.05. Results are mean of three separate experiments.

Motility was measured using semisolid motility agar by measuring the distance moved by the bacteria after 48 h in the presence or absence of NA. The diameter of the motility halo was significantly higher in the presence of NA for all the species of *Campylobacter* (*P* < 0.05; Figure 2).

### 3.3. Effect of Noradrenaline on the Invasion of T84 Cells

**Figure 3:**
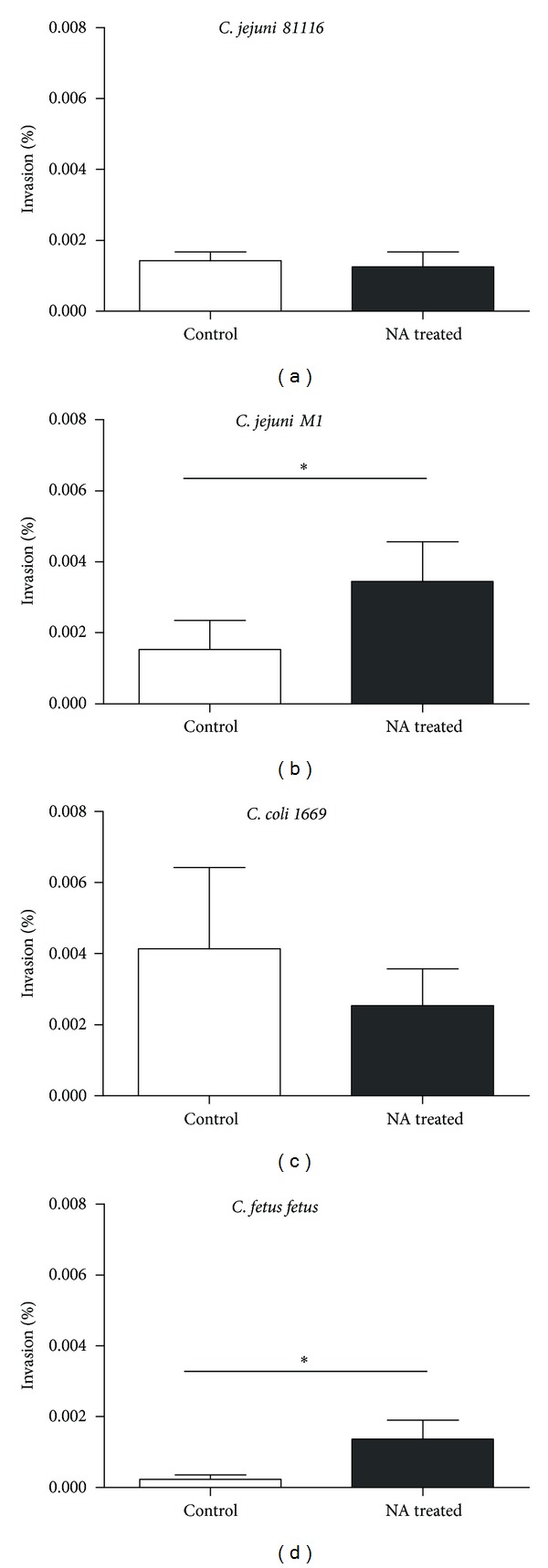
Invasion of T84 epithelial cells by *C. jejuni 81116* (a), *C. jejuni M1* (b), *C. coli* 1669 (c), and *C. fetus fetus* (d) at 37°C was determined by gentamicin protection assay. The percentage of invading cells in the inoculum in the presence (black) or absence (white) of noradrenaline is shown. Results are the mean of three separate experiments, and bars represent standard error. Star indicates *P* < 0.05.

The invasion efficiency of *Campylobacter* species pretreated with NA was analysed using a gentamicin protection assay. In this experiment, *Campylobacter* species were grown in the presence of noradrenaline for 48 hours and used to infect a T84 monolayer for 90 minutes and the number of bacteria invading into cells was counted. There was 4 and 5 times more number of *C. jejuni M1* (*P* = 0.013) (Figure 3(c))* and C. fetus fetus* (*P* = 0.05) (Figure 3(d)), respectively, which were recovered from cells treated with noradrenaline compared to controls. However, there was no significant difference in the invasion of cells by NA-treated *C. jejuni 81116* (Figure 3(a)) and *C. coli* 1669 (Figure 3(b)) compared to the untreated group.

### 3.4. Effect of NA on the Tight Junction Integrity of T84 Cells

**Figure 4:**
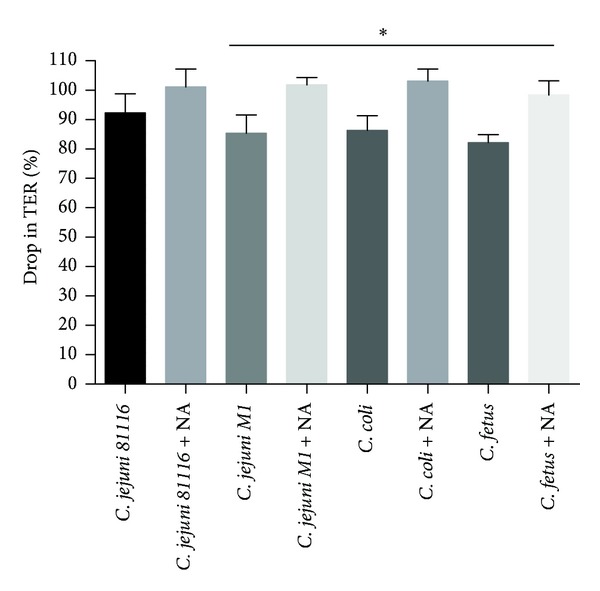
The effect of noradrenaline on the tight junction integrity of T84 epithelial cells was measured by percentage drop in transepithelial resistance (TER) at 48 hours after infection with *Campylobacter* species. Results are the mean of three separate experiments, and bars represent standard error. Star indicates *P* < 0.05. A significantly higher drop in TER was noticed when noradrenaline was added to the transwells infected with *C. jejuni M1*, *C. coli,* and *C. fetus fetus* only.

T84 cells grown on transwells were infected with *Campylobacter* in the presence or absence of NA and tight junction integrity measured by reading TER at T0 and T48 hours. We observed reduction in TER when T84 cells were infected with *Campylobacter*. Furthermore, the reduction in TER increased when noradrenaline was added along with the bacteria. The drop in TER was at least 16% higher (*P* < 0.05) in case of *C. jejuni M1, C. coli, *and *C. fetus fetus* (Figure 4). *C. jejuni 81116* did not show increased breakdown of tight junctions when noradrenaline was added.

**Figure 5:**
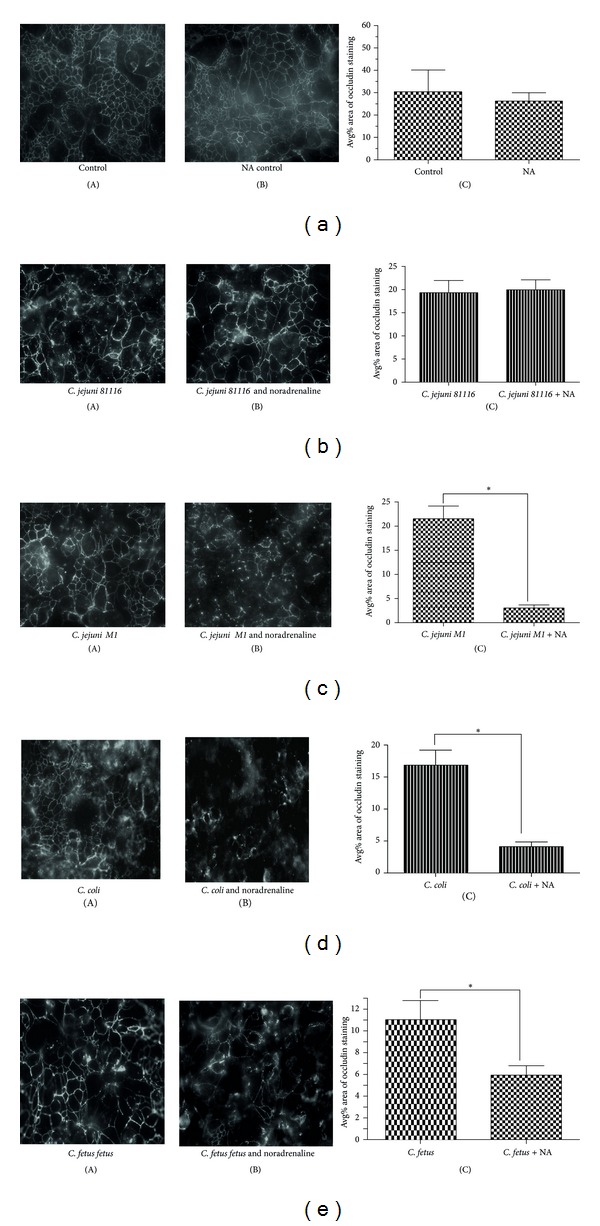
Effect of *Campylobacter* on integrity of T84 monolayers. Monolayers were fixed and stained for tight junction protein occludin after 48-hour infection with *Campylobacter* or *Campylobacter* with NA and analysed by confocal microscopy. Monolayers exposed to *C. jejuni 81116, C. jejuni M1, C. coli*, and *C. fetus fetus* in the absence of noradrenaline shown as control in (b)–(e) (A) (the first figure). Cell monolayers co-infected with *Campylobacter* in the presence of noradrenaline are shown as treated group in (b)–(e) (B) (the second figure). Ten fields of view were visualised using confocal microscopy with a Hamamatsu camera and digitised using Leica Q fluoro software and analysed by quantitative immunofluorescence using Macros on image J software. The average area of percentage area of occludin staining was measured in ten views and compared by an unpaired test ((a)–(e) (C)). Results shown are from a single experiment; *represents *P* value <0.05.

Tight junctions were stained for occludin and microscopic images analysed by measuring the percentage area of staining for this protein from ten field views of a slide. The percentages in each were compared by unpaired *t*-test and the tests were done in triplicate. Figures 5(a)–5(e) show results from a single experiment representative of triplicates. Occludin fragmentation and the loss of honeycomb structure depict tight junction breakdown. After 48 hours, a honeycomb structure in both uninfected T84 monolayers (Figure 5(a)(A)) and uninfected T84 monolayers treated with noradrenaline was observed (Figure 5(a)(B)), indicating the presence of intact junctions. There was no difference in the percentage area of occludin staining between control T84 monolayers and control T84 monolayers treated with noradrenaline (Figure 5(C); *P* = 0.73). We observed higher breakdown of tight junctions in T84 monolayers infected with *C. jejuni M1* (*P* = 0.0001, Figure 5(c)(A), (B), and (C)), *C. coli* (*P* = 0.001, Figure 5(d)(A), (B), and (C)), and *C. fetus fetus* (*P* = 0.014, Figure 5(e)(A), (B), and (C)) in the presence of NA when compared to T84 cells infected with above strains in the absence of noradrenaline. However, noradrenaline did not affect the junction integrity of T84 monolayers when they were infected with *C. jejuni 81116* (Figure 5(b)(A), (B), and (C); *P* = 0.85).

### 3.5. Effect of NA Treatment on the Colonisation of Chickens by *Campylobacter*

**Figure 6:**
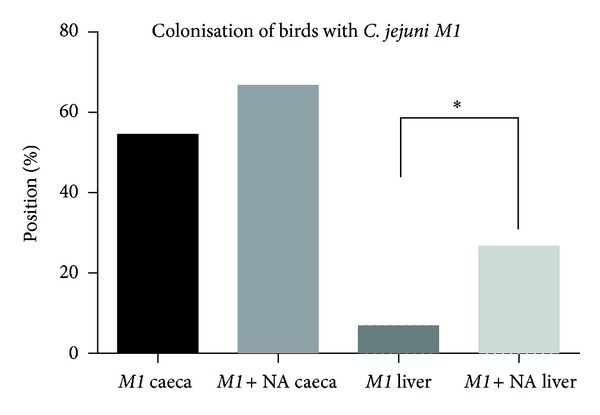
The percentage of caeca and livers positive when chickens were inoculated with *C. jejuni* or *C. jejuni* pretreated with NA. Notice that higher numbers of birds were positive in caecum in NA pretreated group than when infected with *C. jejuni* alone. Higher invasion into livers is seen in the NA-treated group only when infected with *C. jejuni M1*. Results are from a single experiment consisting of ~26 birds in a group. Statistical difference was assessed by a chi-square test and star represents significant *P* < 0.05.

Chicks were given* C. jejuni M1* cultures grown in the presence or absence of NA and after 48 hour; caecum and liver samples were examined for the number of *Campylobacter* present. Growth in the presence of NA did not significantly affect the ability of *C. jejuni M1* to colonise chicks. Thus, 84% of caeca from birds given control cultures were *Campylobacter* positive compared to 96% given NA-treated cells. However, NA-treated cells of *C. jejuni M1* were better able to leave the intestinal tract and 42% of liver samples from birds given these bacteria were *Campylobacter* positive compared to only 12% in birds given control cultures (*P* = 0.008; Figure 6). The CFU/g of bacteria in liver tissue was calculated (livers positive on enrichment were given a value of 0.5). The infection level in livers from birds given NA-treated *C. jejuni* was 354 CFU/g but only 0.05 CFU/g (*P* = 0.004) in birds given the control *Campylobacter* culture.

## 4. Discussion

*Campylobacter* species are the major causative agents of gastroenteritis in humans. The understanding of their behavior in the food chain is vital for effective control in food production to reduce the risk of transmission. Cogan et al. noticed an increase in growth rate, motility, and invasion by *C. jejuni 11168* following exposure to noradrenaline [20]. In the current study, we explored the effect of NA *in vitro* and *in vivo* on prevalent species of *Campylobacter*. In contrast to the previous study, which examined the effects following preexposure of bacteria to NA, we studied these effects in the presence of noradrenaline. Iron is an essential nutrient and acts as a cofactor for many enzymes involved in essential cellular processes, including electron transfer [28]. Enterochelin is a siderophore produced by most genera of enteric bacteria to chelate iron from transferrin [29]. *Campylobacter* has not been shown to produce siderophores [30] but has been shown to utilise enterochelin [30]. Noradrenaline has catechol moiety similar to enterochelin and is suggested to sequester iron from transferrin/lactoferrin and supply for bacterial growth [31, 32]. Noradrenaline was suggested to facilitate iron uptake by* C. jejuni 11168* in the presence of serum [20]. In this study, a tissue environment low in iron was simulated by using DMEM supplemented with 10% FCS. In this medium, NA was found to reduce the lag phase and increase the growth and the final optical density for most of the *Campylobacter* species tested. Freestone et al. noticed a 10-fold or greater increase in growth of 15/17 gram-negative and 4/6 gram-positive bacteria when media were supplemented with NA in iron limited conditions [31]. Noradrenaline also increased the growth of *E. coli*, *Pseudomonas,* and *Yersinia* [11]. *E. coli* strains, which failed to grow in SAPI + serum medium (minimal medium supplemented with 30% serum) at 10^2^ CFU/mL, responded to 50 *μ*M NA by reaching a final population density of 10^8^ CFU/mL [29]. The precise underlying mechanism for increased growth when *Campylobacter* was treated with NA is not clear. Freestone et al. proposed that serum (transferrin) in the DMEM medium binds iron and NA is proposed to facilitate the transfer of iron from transferrin and similar host iron-binding proteins to bacteria, thus stimulating microbial growth in iron-restricted environments [32].

A 76-kDa protein, CfrA, was recently characterized as the potential iron-siderophore transporter in *C. jejuni* [33]. Zeng et al. suggested that NA facilitates iron uptake in *C. jejuni 11168* through a siderophore-mediated process via the enterochelin receptor CfrA [34]. The *C. jejuni 81116* genome contains a single gene encoding TonB and lacks the CfrA and Cj0178 outer membrane receptors for iron uptake [35]. A Blast search of the *cfrA* gene also revealed its absence in* C. jejuni M1*. The results of these experiments have shown that all species of *Campylobacter jejuni* have increased growth in the presence of noradrenaline. This suggests that *C. jejuni 81116* and *C. jejuni M1* may have a CfrA homologue, which is responsible for noradrenaline-mediated effects. Zeng et al. proposed a phylogenetic tree of the *cfrA* gene and divided it into two main groups; *C. jejuni 11168* was present in one group and *C. jejuni 81116* in the other and there was 93 to 98% amino acid similarity for *cfrA* in between the groups [34]. Taken together, it can be suggested that CfrA is responsible for noradrenaline-mediated growth promotion in *C. jejuni* and a homologue of CfrA in some species of *C. jejuni* can contribute to the same effect.

Motility is an important factor for invasion; mutants in flagella export apparatus have shown reduced colonisation in chicks and decreased invasion of Caco-2 cells [36]. The swimming characteristic of *C. jejuni* in viscous environments may be an important factor in the interaction with host epithelial cells [37]. Cogan et al. found that ~18% of *C. jejuni* cells were motile in a low iron medium compared to ~60% in one containing NA, suggesting that the neurotransmitter can increase the number of motile and potentially invasive cells and allow better colonisation of the intestine [20]. The motility-related genes *fliA, fliY, fliB*, and *flhC* were significantly upregulated when *Salmonella* was exposed to NA [18]. The results of this study also showed higher motility in the presence of NA (Figure 2). However, it is not known if the motility genes are up regulated in the presence of NA in *Campylobacter* spp. and this should be investigated.

Cogan et al. examined invasion of Caco-2 cells by *C. jejuni 11168* and noticed that at least 10 times more bacteria were recovered from within epithelial cells after 2 hours when they were pretreated with NA [20]. In these experiments, when T84 cells were infected with NA pretreated *C. jejuni M1* and *C. fetus fetus*, four to five times as many bacteria were recovered within 90 minutes of inoculation (Figure 3). These observations suggest that pretreatment of *Campylobacter* with NA makes them more invasive, though some strain differences are seen. *C. jejuni 81116* and *C. coli* did not show significantly increased invasion of T84 cells. This may be because the invasion time of 90 minutes was not long enough to facilitate invasion by these strains or because pretreatment with NA had no/minimal effect on their virulence.

*C. jejuni* invasion causes loss of tight junction barrier function, which can be indicated by the significant fall in TER and occludin redistribution from the junctional platform to the cytosolic compartment [38]. In these experiments, when T84 cells were infected with *C. jejuni M1*, *C. coli*, and *C. fetus fetus* (Figure 4), there was a reduction in TER, which increased when NA was added along with the bacteria (Figure 4). *C. jejuni 81116* did not show any increased breakdown of tight junctions when NA was added. Cogan et al. found that, when a Caco-2 monolayer was infected with* C. jejuni 11168* grown in 100 *μ*M noradrenaline, there was approximately a 50% reduction in TER within 24 hours and occludin staining was fragmented, indicating disruption of the tight junctions between cells. In this work, when a T84 monolayer was infected with *Campylobacter* species along with NA, there was approximately 100% drop in TER and a complete disruption of tight junctions within 48 hours suggesting the increased virulence of *Campylobacter* in the presence of noradrenaline. Chen et al. observed that NA increased mucosal adherence of wild-type *E. coli* O157:H7 strain 85–170 in porcine caecal mucosa [13]. This effect occurred within 30–90 minutes after bacterial inoculation, which suggests that NA can modulate early, nonintimate adherence leading to invasion [13]. Lyte et al. have noted an increase in the expression of K99 pilus adhesion of enterotoxigenic *E. coli in vitro* following exposure to NA [39]. These observations suggest that noradrenaline-mediated increased invasion may be due to increased adhesion of bacteria to the epithelium. It is not known if NA causes any changes in the expression of virulence genes and there is a need to explore the exact reason for the breakdown of tight junctions.

*Campylobacter* contamination of chickens is a major public health concern [40]. Chicken colonisation experiments were performed to understand the kinetics of poultry colonisation, which can help us find measures to decrease the risk of campylobacteriosis to public health. *C. jejuni M1* has shown higher colonisation of birds in the NA-pretreated group. Invasion into livers occurred in more birds in the NA-pretreated group. Furthermore, the number of bacteria isolated from the positive livers was also significantly higher (Figure 6) suggesting increased virulence of *Campylobacter* on pretreatment with noradrenaline. *Campylobacter* pretreated with NA simulates the *Campylobacter* shed from a stressed animal, which is now shown to be more virulent and invasive in a shorter period.

Taking the conclusions drawn from these observations into the field, transport of chickens causes the birds to be stressed and *Campylobacter* to be shed from the birds. The *Campylobacter* will have been exposed to NA and will be shed at a higher rate (due to increase in growth) and be more virulent and invasive (due to higher motility and invasion) to other chickens causing increased contamination of the flock and subsequently the carcasses. This may suggest the reason for flocks negative at the farm level becoming colonised after transport and holding [41–44]. So taking measures such as reducing the transport times, causing less stress to birds during, transport may reduce the spread and prevalence of *Campylobacter* on carcass.

To conclude, it appears that NA increases the growth and virulence of *C. jejuni*, allowing better colonisation and invasion of the avian host and potentially increasing the risk of transmission of *Campylobacter* to humans. Hence, the presence of NA, brought about by acute stress in humans, may thus allow greater multiplication and increased virulence of *Campylobacter* in the host. This may contribute to the range of symptoms of *Campylobacter* infection, ranging from asymptomatic infection to serious complications, seen in different individuals. Stress-associated hormones appear to contribute to the virulence associated properties of diverse *Campylobacter* species.

## Figures and Tables

**Table 1:** The OD_600_ values at the start of stationary phase of growth in the presence and absence of NA at 37°C. The mean ± SEM of three experiments was compared using an unpaired *t*-test and the significance values (*P* value) are shown.

OD_600_ values at 37°C	Control	+NA	*P* value
	Mean ± SEM	Mean ± SEM	
*C. jejuni 81116*	0.0310 ± 0.0130	0.0802 ± 0.0061	0.0135
*C. jejuni M1*	0.0358 ± 0.0165	0.0951 ± 0.0154	0.0290
*C. coli 1669*	0.0767 ± 0.0009	0.1263 ± 0.0137	0.0112
*C. fetus fetus 10842*	0.0022 ± 0.0006	0.0692 ± 0.0023	0.0001
*C. coli RM2228*	0.0902 ± 0.0042	0.1273 ± 0.0075	0.0062
*C. jejuni 11168*	0.0497 ± 0.0052	0.0955 ± 0.0023	0.0006
